# Book Reviews

**Published:** 1893-01

**Authors:** 


### Bibliographical.

A TREATISE ON NERVOUS AND MENTAL DISEASES: For the use of Students and Practitioners. By Landon Carter Gray, M. D., Professor of Nervous and Mental Diseases in the New York Polyclinic. In one very handsome octavo volume of 681 pages, with 168 illustrations. Cloth, $4.50; leather, $5.50.

FROM THE PREFACE.

This book is an endeavor to put a working knowledge of nervous and mental diseases at the command of students and practitioners. It is addressed especially to the general practitioner, present or future, and its theme is rigidly therapeutical. Those who may use it will find that it does not assume previous knowledge of its subjects on their part. The space assigned to the several diseases is determined by the needs of the profession at large, a consideration which has made the chapter on Neurasthenia the largest in the book. Mental diseases are considered from the standpoint of the general physician, since the outcome will often depend on his skill in early recognition and treatment. I have endeavored to indicate what diseases of the mind may be best treated at home, what may need both home and asylum treatment, and the period of committal, and what may require seclusion from the outset. A novel feature in a work of this kind is the inclusion of the medico legal aspects of nervous and mental diseases. In carrying out the therapeutic aim of the book, each chapter has been made to contain its own section on treatment. Especial care has been taken to make the therapeutical suggestions sufficiently precise to cover the varying stages, symptoms, and complications of disease. In the chapters upon Mental Diseases the most approved treatment is stated, and likewise the results to be expected. Only that knowledge has been admitted to these pages which has stood the test of experience. Students will find the terminology rendered easy of acquisition by the derivations and definitions given in the Glossary. Throughout the volume simple Anglo-Saxon terms and familiar synonyms have been preferred. Those who are desirous of further investigation will find a full bibliography appended to each chapter. I have made liberal use of illustrations, deeming them essential for explanation and vividness.

---

THE NATIONAL MEDICAL DICTIONARY.—Including English, French, German, Italian and Latin Technical Terms Used in Medicine and the Collateral Sciences, and a Series of Tables of Useful Data. By John S. Billings, M. D., LL. D., Edin. and Harv.; D. C. L., Oxon., Member of the National Academy of Science, Surgeon U. S. A., etc. In two very handsome royal octavio volumes containing 1574 pages, with two colored plates. Per volume: Cloth, $6.00; leather, $7 00; half Morocco, marbled edges, $8.50. For sale by subscription only. Specimen pages on application. Address the Publishers.

The work is remarkable for its fullness. The enumerations and subdivisions under each word-heading are strikingly complete as regards alike the English tongue and the languages chiefly employed by ancient and modern science. It presents to the English reader a thoroughly scientific mode of acquiring a rich vocabulary and offers an accurate and ready means of reference in consulting works in any of the three modern continental languages which are richest in medical literature. To add to its usefulness as a work of reference some valuable tables are given. Another feature of the work is the accuracy of its definitions. One is enabled by the work under notice to read intelligently any technical treatise in any of the four chief modern languages. There cannot be two opinions as to the great value and usefulness of this dictionary as a book of ready reference for all sorts and conditions of medical men So far as we have been able to see, no subject has been omitted, and in respect of completeness it will be found distinctly superior to any medical lexicon yet published.—*The London Lancet.*

